# Development of β-cyclodextrin/polyvinypyrrolidone-co-poly (2-acrylamide-2-methylpropane sulphonic acid) hybrid nanogels as nano-drug delivery carriers to enhance the solubility of Rosuvastatin: An in vitro and *in vivo* evaluation

**DOI:** 10.1371/journal.pone.0263026

**Published:** 2022-01-21

**Authors:** Hina Shoukat, Fahad Pervaiz, Mehran Khan, Sadia Rehman, Faizan Akram, Usman Abid, Sobia Noreen, Muhammad Nadeem, Rubina Qaiser, Rizwan Ahmad, Irshad Farooq

**Affiliations:** 1 Faculty of pharmacy, Department of Pharmaceutics, The Islamia University of Bahawalpur, Bahawalpur, Pakistan; 2 Department of Pharmacy, Bahauddin Zakariya University, Multan, Pakistan; 3 Shalamar Hospital Lahore, Lahore, Pakistan; Central University of Rajasthan, INDIA

## Abstract

The present study is aimed at enhancing the solubility of rosuvastatin (RST) by designing betacyclodextrin/polyvinypyrrolidone-co-poly (2-acrylamide-2-methylpropane sulphonic acid) crosslinked hydrophilic nanogels in the presence of crosslinker methylene bisacrylamide through free-radical polymerization method. Various formulations were fabricated by blending different amounts of betacyclodextrin, polyvinylpyrrolidone, 2-acrylamide-2-methylpropane sulphonic acid, and methylene bisacrylamide. The developed chemically crosslinked nanogels were characterized by FTIR, SEM, PXRD, TGA, DSC, sol-gel analysis, zeta size, micromeritics properties, drug loading percentage, swelling, solubility, and release studies. The FTIR spectrum depicts the leading peaks of resultant functional groups of blended constituents while a fluffy and porous structure was observed through SEM images. Remarkable reduction in crystallinity of RST in developed nanogels revealed by PXRD. TGA and DSC demonstrate the good thermal stability of nanogels. The size analysis depicts the particle size of the developed nanogels in the range of 178.5 ±3.14 nm. Drug loading percentage, swelling, solubility, and release studies revealed high drug loading, solubilization, swelling, and drug release patterns at 6.8 pH paralleled to 1.2 pH. *In vivo* experiments on developed nanogels in comparison to marketed brands were examined and better results regarding pharmacokinetic parameters were observed. The compatibility and non-toxicity of fabricated nanogels to biological systems was supported by a toxicity study that was conducted on rabbits. Efficient fabrication, excellent physicochemical properties, improved dissolution, high solubilization, and nontoxic nanogels might be a capable approach for the oral administration of poorly water-soluble drugs.

## 1. Introduction

Recently, advances in combinatorial chemistry and biotechnology have headed to the discovery of a wide range of novel chemical compounds with therapeutic potential. As with many existing drugs, the majority of these recently discovered molecules and their derivatives are hydrophobic by nature. The main issue that has arisen as a result of this is its little water solubility and, as a result, its limited bioavailability [1,2]. A well-known fact is the three main factors that influence drug absorption and bioavailability namely solubility, dissolvability, and intestinal permeability. Orally administered drugs’ aqueous solubility is a critical factor in their absorption [3]. Owing to their less solubility and minute dissolution velocity, poorly hydrophilic drugs present a less concentration gradient among the GIT (gastrointestinal tract) and the blood vessels leading to imperfect transport and ultimately affecting oral absorption [4]. Drugs must be water soluble in order to elicit desirable effects at low doses because water makes up the majority of the human body. For that purpose, scientists are attempting to increase the solubility and bioavailability of lipophilic drugs [5,6].

Hydrophobic and hydrophilic drugs can both be encapsulated in nanogels, which are polymer-based nano-drug delivery systems [7]. Nanogels are hydrogels that have been scaled down to nanometer-sized dimensions. Heteropolymer network with high crosslink density that has the tendency to absorbs and hold large amounts of water without changing its internal network structure [8–10]. In addition to their smaller size and surface area, nanogels also have a high drug-loading capacity and high stability, making them a novel, safe and effective carrier system for drugs with low solubility. As a result of their extra-small size, they are very promising in drug delivery applications [11,12]. Moreover, due to low entrapment efficacy, nanoparticles have limited use. To overcome the drawback of nanoparticles, cyclodextrin has been incorporated into nano polymeric networks. Polymeric biomaterials that host active moiety have increased loading capacity [13].

The acyclic oligosaccharide-β-Cyclodextrin (β-CD) has a hydrophilic outer rim and a hydrophobic inner chamber [14]. CDs are utilized in a variety of industries, as they have the inherent capacity to enhance the physicochemical characteristics of drugs through complexation. After CDs are polymerized with other polymers to form gels, their capability to increase solubility as well as their bioavailability. In the existence of strong sulfonic acid and aqueous media, the Ritter reaction yields AMPS, a white crystalline powder made from acrylonitrile and isobutylene (water). Pharmaceutical drug carriers based on AMPS dissociate across all pH ranges, causing pH-independent swelling. AMPS-based gels can also be used in skin-sensitive electrodes, biomedical engineering carriers, muscle actuators, and medication delivery [15,16].

RST (rosuvastatin calcium) is a low-solubility, high-permeability BCS class II medication. It’s a water-insoluble inhibitor of 3-hydroxy-3-methyl glutaryl CoA (HMG-CoA) reductase. This enzyme catalyses the initial and rate-controlling step in cholesterol formation, the conversion of HMG-CoA to mevalonate, a powerful lipid lowering component and hypolipidemic medication. It is more potent among other statins as it is not metabolized by cytochrome P450 3A4 and less potential for drug drug interaction [17,18]. It is used to treat familial hyperlipidemia, triglyceridemic disorders, dyslipidemia, osteoporosis, atherosclerosis, benign prostatic hyperplasia, and Alzheimer’s disease. Due to its poor solubility in water and significant processing provided by the liver through oxidation, lactonization, and glucuronidation, it has a limited bioavailability of 20% [19].

We aimed to introduce a new concept of using hydrophilic polymers and monomers as fundamental excipients in the development of chemically cross-linked nanogels that would improve drug solubility and accelerate drug release. β-CD, PVP, and AMPS polymeric network systems were successfully developed, tuned, and optimized to improve the solubility of rosuvastatin. These systems have potential advantages over previously reported carrier systems in terms of drug release, solubility, and crystallinity. Polymer, monomer, and crosslinker concentrations were varied to create hydrogel nanoparticles that were optimized for a specific application. The approach can be thought of as a straightforward way for making nanogels via chemical cross-linking of N,N′-methylene bisacrylamide (MBA) that has been tweaked and optimised for solubility increase. This hydrogel nanoparticulate technology to improve RST calcium solubility is not reported previously.

## 2. Materials and methods

### 2.1. Materials

Rosuvastatin was provided as a gift by SAMI pharmaceuticals (Pvt.) Limited. Karachi, Pakistan. β-cyclodextrin (β-CD), Polyvinylpyrrolidone, Ammonium peroxodisulphate, Methylenebisacrylamide (MBA) were acquired from Sigma- Alddrich, UK. 2-acrylamido-2-methyl propane sulphonic acid was procured from Shouguang Pner Chemical Co; Ltd, China.

### 2.2 Methodologies

#### 2.2.1. Synthesis of nanogels

Fabrication of nanogels was carried out through free radical polymerization technique with slight modification of previously reported methods [6–20]. The synthetic process involves the use of different proportions of polymer, monomer, and crosslinker to determine the influence of varying concentrations. The content of used ingredients in the fabrication of nanogels is reported in Table 1.

**Table 1:** Composition of βCD/PVP-co-poly (AMPS) nanogels.

	Code of formulation	Concentration (g) of nanogel formulations ingredients per 20 g of distilled deionized water				
		βCD	PVP	AMPS	APS	MBA
**1**	BPAE-1	0.1	0.1	3	0.1	1g
**2**	BPAE -2	0.1	0.1	4	0.1	1g
**3**	BPAE -3	0.1	0.1	5	0.1	1g
**4**	BPAE -4	0.1	0.1	4	0.1	2g
**5**	BPAE -5	0.1	0.1	4	0.1	3g
**6**	BPAE -6	0.1	0.08	4	0.1	1g
**7**	BPAE -7	0.1	0.12	4	0.1	1g
**8**	BPAE-8	0.2	0.1	4	0.1	1g
**9**	BPAE-9	0.3	0.1	4	0.1	1g

* βCD (Betacyclodextrin), PVP (Polyvinyl pyrrolidone), AMPS (2-acryl amido-2-methyl propane sulphonic acid), APS (Ammonium peroxodisulfate), MBA (Methylene bisacrylamide).

**Fig 1:**
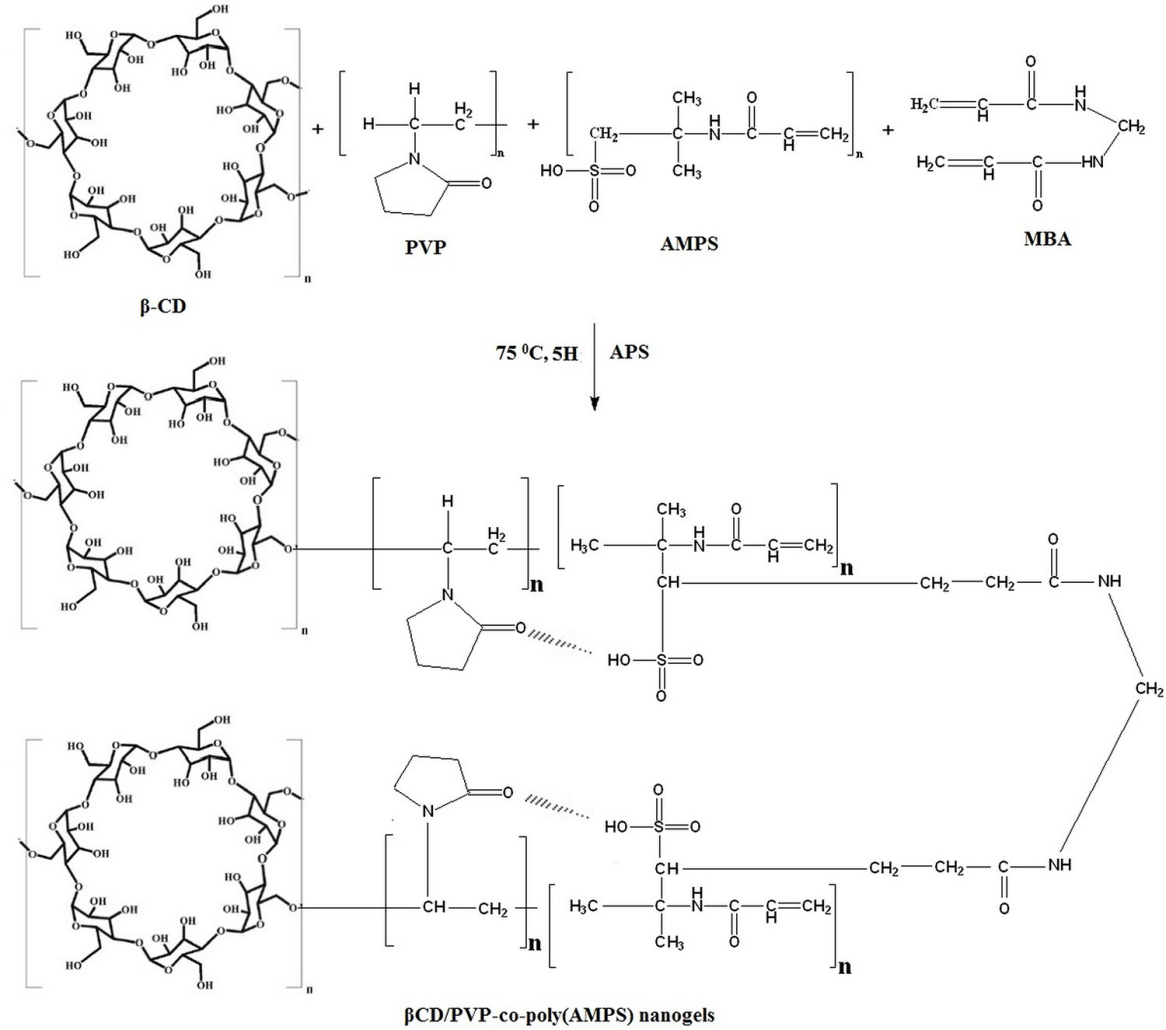
Schematic illustration of βCD/PVP-co-poly (AMPS) nanogels showing possible chemical crosslinking.

The aqueous solutions of PVP, APS, and AMPS were prepared separately. Weighed concentration of βCD was completely dissolved in a water-ethanol mixture (1:1) with aid of heat. Similarly, a specified content of crosslinker (MBA) was dissolved in a water-ethanol mixture(1:2) at a temperature of 60°C. Initiators (APS) solution was included in monomer (AMPS) solution and agitated for 5–10 minutes to initiate the polymerization reaction. Initiator and monomer solutions were added to the polymer solution of βCD and PVP upon stirring to obtain a homogenous mixture. Finally, added the clear MBA solution to the above mixture and stirred for 2–3 minutes. After stirring, poured the clear solution into a round bottom flask, placed it in the water bath, and fitted with a condenser. The water bath’ temperature was set at 75°C. Subsequently, 4–5 hr, a gel type structure was formed which was sieved to attain nanogels. Water and ethanol mixture (1:1) was used for washing of nanogels which were then placed in oven at 40°C until dry. Rosuvastatin was loaded in developed nanogels by preparing drug (RST) solution. About 1% RST solution was prepared in a mixture of methanol and distilled water (4:1) with a final volume of 100 mL due to solubility concerns of RST. Specified quantity (0.5 g) of developed nanogels was placed in the prepared RST solution and kept on sonication for half-hour at room temperature and then lyophilized for 4–5 h and characterized. A schematic illustration of prepared nanogels is shown in Fig 1.

## 3. Characterizations of βCD/PVP-co-poly (AMPS) nanogels

Prepared nanogels were characterized and formulation BPAE-3 was selected for characterization due to good stability and release properties.

### 3.1. Fourier transform infrared spectroscopic (FT-IR) analysis

To confirm the formation of βCD/PVP-co-poly (AMPS) nanogels, FT-IR spectra of pure drug rosuvastatin, βCD, PVP, AMPS and drug loaded nanogels (BPAE-3L) formulations were carried out using Fourier transform infrared (FT-IR) spectrophotometer (Tensor 27 series; Bruker Co. Germany) over the range of 400–3500 cm^−1^ [21].

### 3.2. Morphological characterization

The surface morphology of drug loaded nanogels was analyzed at various resolutions by means of scanning electron microscopy SEM (JSM-6490A, Tokyo, Japan). To examine the porosity and proper shape of nanogels, sample fabricated for SEM evaluation were attained with lyophilization and mounted on the gold-coated aluminum stubs using carbon adhesive tape and examined [21,22]

### 3.3. Powder X-ray diffractometry (PXRD)

Powder X-ray diffractometry (PXRD) was carried out to define the amorphous or crystalline attributes of the drug (RST), polymers (PVP, βCD), monomers (AMPS), and unloaded and RST loaded nanogels as amorphous nature of individual moieties and the delivery system is favored to profound bioavailability and enhanced aqueous solubility [23].

### 3.4. Particle size analysis

Analysis of particle size of fabricated βCD nanogels is executed by Zetasizer (Malvern Instruments, UK) as solubility is intrinsically related to particle size reduction because a decline in particle size rise the surface area to volume ratio, hydrophobic medicines have better solvation and solubility. Moreover, the system’s stability was evaluated by the value of zeta potential [13].

### 3.5. Micromeritics properties

The micromeritics properties of all fabricated formulations were calculated including the bulk density, angle of repose, tapped density, Hausner’s ratio and Carr’s compressibility index. All the results were calculated with formulas given below [24–28];

$$Angle\;of\;repose=Tan\;Ɵ=\frac{h}{r}$$ (1)

Where, h = height of blend, and r = radius of base

$$Bulk\;density=\frac{Mass}{bulk\;volume}$$ (2)

$$Tapped\;density=\frac{Mass}{tapped\;volume}$$ (3)

$$Carr’s\;index=\frac{tapped\;density-bulk\;density}{tapped\;density}\times 100$$ (4)

$$Hausner\;ratio=\frac{tapped\;density}{bulk\;density}$$ (5)

### 3.6. Thermal analysis

Thermal analysis of βCD, PVP, AMPS, RST, unloaded and drug loaded βCD/PVP-co-poly (AMPS) nanogel were performed by the thermogravimetric analyzer (SDT Q 600 series). The sample was analyzed in triplicate at the rate of 20°C/min below nitrogen stream at the flow rate of 10 ml/min by placing the sample in a platinum pan attached with microbalance [23].

### 3.7. Sol-gel fraction

A sol-gel study was carried out to analyze constituents that were not crosslinked during the fabrication of nanogels. The method selected for sol-gel analysis was Soxhlet extraction. A specified quantity of nanogels was added to the round bottom flask containing 50 ml of deionized water fitted with a condenser. Apparatus was placed in the water bath and the extraction process was continued for 4–5 h at a temperature of 85–90°C. Nanogels were removed and dried for 24–72 h at 40°C till a persistent weight was acquired [23].

Following equations were employed to determine sol and gel fractions.


$$Sol\;fraction(\%)=\left[\frac{W_{1}-W_{2}}{W_{1}}\right]\times 100$$ (6)

Gelfraction(%)=100‐Solfraction (7)

Where W_1_ and W_2_ represents the nanogels’ weight before and after extraction respectively.

### 3.8. Solubility studies

Solubility experiments of developed nanogels in distilled water and buffer solutions maintained at pH 1.2 and 6.8 were used to establish the extent of hydrophobic drug solubility increase. Excess amounts of pure rosuvastatin were put into beakers containing a pre-defined medium, and the beaker was mechanically shaken for 24 hr. The same procedure was used to make nanogels in distilled water and pH 1.2 and 6.8 buffer solutions, which were subsequently exposed to drug solution and mechanical stirring for 24 hr. After 24 hr, supernatant layers from all solutions were collected, filtered, and examined using a UV–Visible spectrophotometer with a maximum wavelength of 243 nm [5].

### 3.9. Swelling analysis

The effect of medium on nanogel’s swelling behavior was established by measuring dynamic swelling at various time intervals (t) with a dialysis membrane that resembled a biological membrane. Specified quantity (100 mg) of nanogels was sealed in dialysis membrane and immersed in 100 ml of 0.5 M buffer solution maintained at pH 1.2 and 6.8. Dialysis membrane (containing 100 mg nanogels) were removed at predefined time intervals of 0, 5, 10, 15, 20, 30, 45, 60, 90, 120 minutes. Surface media was properly cleaned by blotting with Whatmann’s filter paper and membrane were hanged until no liquid drop was oozing from dialysis membrane each time and weight of swollen particles was noted. Dialysis membrane were immersed again in respective pH solution after each reading [19–29]. The dynamic swelling (q) was estimated by using the equation below [5]:

$$q=\frac{W_{2\;}}{W_{1}}$$ (8)

Where W_1_ exhibits the initial weight of nanogel and W_2_ is the final weight of nanogels in swollen state at a given time (t).

### 3.10. Percentage water absorption analysis

The precisely weighed amount of fabricated nanogels (0.2 g ± 0.001 g) was immersed in 200 ml distilled water for 24 hr. The temperature of the soaked nanogel system was kept at 37°C as proposed by previous literature [30–33]

$$Percent\;water\;absorption=\frac{mass\;of\;particles\;after\;soaking-mass\;of\;particles\;before\;soaking}{mass\;of\;particles\;before\;soaking}\times 100$$ (9)

### 3.11. Drug loading and Drug loading efficiency

Rosuvastatin was loaded in developed nanogels by preparing drug (RST) solution in a mixture of methanol and distilled water (4:1). About 1% RST solution was prepared in a mixture of methanol and distilled water (4:1) with a final volume of 100 mL due to solubility concerns of RST. Specified quantity (0.5 g) of developed nanogels was placed in the prepared RST solution and kept on sonication for half-hour at room temperature and then lyophilized for 4–5h. Extraction method was used to determine the drug loading efficiency. Developed nanogels were immersed into poured into phosphate buffer of 6.8pH to assist swelling and drug release. Finally absorbance was taken at the wavelength of 243 nm using a UV–Visible spectrophotometer (Memmert, Germany) and percentage drug loading was calculated by given formula [19].

Percentage drug loading was calculated by given formula;

$$Percentage\;drug\;loading=\frac{Actual\;drug\;content\;in\;nanogels}{Theoretical\;drug\;content\;in\;nanogels}\times 100$$ (10)

### 3.12. *In-vitro* drug dissolution studies

*In-vitro* drug release experiments were performed at different media (pH 1.2 and pH 6.8) to evaluate the best suited media for significant drug release. The in-vitro drug release profile of developed nanogels was performed in 6 stations dissolution test apparatus (USP Dissolution Apparatus II) using 900 ml of phosphate buffer solution at pH 1.2 and pH 6.8 maintained at 37 ± 0.1°C. The 5ml of samples solution was removed at the selected intervals of time (0, 5, 10, 15, 30, 45, 60, 90 and 120 mins) and release of RST was quantified at the wavelength of 243 nm using a UV–Visible spectrophotometer (Shimadzu, Japan) [13–36].

### 3.13. Toxicological studies

Toxicity studies were performed after reviewing the protocols and getting approval by Pharmacy Animal Ethics Committee (PAEC),faculty of pharmacy, the Islamia University of Bahawalpur, Punjab, Pakistan (approval No. 09–2021/PAEC, dated 8th March 2021), to determine the safety profile of developed nanogels. Twelve healthy rabbits with an average weight of 1.5–2.0 kg were distributed into two groups; group I was defined as the control group while group II was marked as nanogels treated group. Both groups were given access to water and food while developed nanogels were only fed to group II, limiting the content of nanogels at 5 g/kg of rabbits’ body weight. Strict observations were made for both groups. Characteristics of rabbits like weight, water consumption, physical examination, food consumption, and mortality rate were keenly observed. Blood biochemistry and histopathological examination of organs were keenly perceived by withdrawing the blood sample and collecting the organ of sacrificed animals respectively after the 14th day of quarantine. One way ANOVA was applied to calculate the p-value [23].

### 3.14. Hemolysis assay

Hemolysis assay was conducted followed by the guidelines given by ASTM (American society for testing materials) with few modifications [37]. Briefly, the blood samples were collected in citrated blood collection tubes by healthy white New Zealand Rabbits after getting approval by Pharmacy Animal Ethics Committee (PAEC), faculty of pharmacy, the Islamia University of Bahawalpur, Punjab, Pakistan (approval No. 09–2021/PAEC, dated 8th March 2021). A sample of 0.5 ml of blood (10 g/dl hemoglobin) was added to 3.5 ml of nanogel solution and incubated at 37°C for 3 h. The nanogel solution was formed by using phosphate buffer as solvent with concentration of 0.5 mg/ml, 0.3 mg/ml, and 0.1 mg/ml. The incubated tubes were inverted after every half hour to homogenize the mixture. Distill water and buffer were used as positive control and negative control, respectively. Then, centrifugation of the suspension was performed for 15 min and 0.5 ml of the above supernatant was added to 0.5 ml of Drabkin’s reagent and placed at room temperature for 15 min. Finally, the absorbance was taken at 540 nm [38].

### 3.15. *In vivo* pharmacokinetic studies

To examine the pharmacokinetics of drug-loaded fabricated nanogels, twelve white New Zealand Rabbits, weighing 1.5–2.5 kg on average, were divided into two groups; Group A received a single dosage of nanogels adding 5 mg of RST calcium, while Group B received RST commercially available 5 mg Rovista tablets (Getz Pharmaceuticals Karachi, Pakistan). From the jugular vein, samples were withdrawn at a scheduled interval (0, 0.5, 1, 1.5, 2, 3, 4, 8, and 12 hr) for examination. The Pharmacy Research Ethics committee reviewed and approved the protocols of the study (approval No. 09–2021/PAEC, dated 8th March 2021). Evaluation of extracted sample was done by already reported HPLC–U.V. method [39]. Centrifugation for fifteen minutes at 3000 rpm was carried out to separate the plasma. 1 mL of extracting solvent (ethyl acetate) was mixed to 100 μL of above extracted plasma along with 100 ng/mL of Rosuvastatin. Then vortexed the resulted samples for five minutes, centrifuged for ten minutes at 4000 rpm and finally evaporation or remaining supernatant was performed under nitrogen stream. Reconstitution of dried sample was done with 0.3ml of mobile phase consisted of water, methanol and acetonitrile in 1:4:6. Orthophosphoric acid was used to adjust the pH of mobile phase at 5.5. 20 μl sample was injected in HPLC and C18 column was used for analysis. The mobile phase was run at a flow rate of 1.0 ml per min and the UV detector was set at 243 nm. The analytical data of HPLC were plotted as RST concentration (ng/mL) versus time (h). Software package Kinetica v 4.4 was applied to carry out the noncompartmental pharmacokinetic analysis [39].

### 3.16. Stability studies

ICH guidelines were followed to while conducting stability studies of rosuvastatin loaded and. Fabricated nanogels packed in glass vials were kept in stability chamber (Memmert Beschickung, Japan) at 75 ± 5% RH with 40 ± 2°C. After time period of 0, three and six month, sampling was carried out. Developed gels were observed for physical variation, percentage of drug loading and solubilization efficiency [29].

### 3.17. Statistical analysis

Software package Kinetica v 4.4 was applied to carry out the noncompartmental pharmacokinetic analysis. All data were represented as mean ± SD between experiments. Statistical analysis were done by ANOVA and P-value less than 0.05 were considered as significant.

## 4. Results and discussion

### 4.1. FTIR spectroscopy

**Fig 2:**
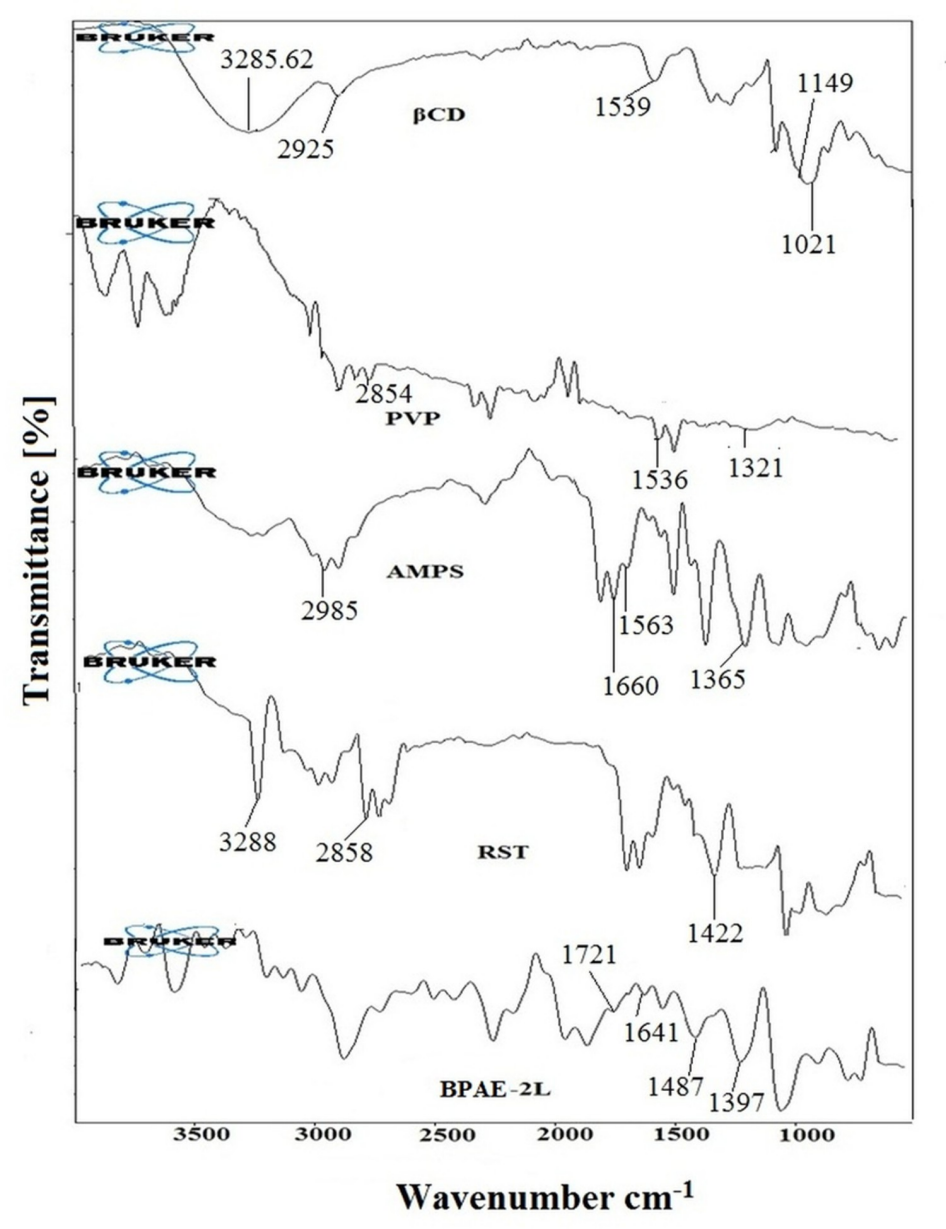
FTIR spectra of βCD, PVP, AMPS, rosuvastatin and loaded (PBCD-2L) fabricated nanogels indicating the peak shifting due to crosslinking.

FTIR spectra of pure drug rosuvastatin, βCD, PVP, AMPS, and developed RST-loaded nanogels formulations were recorded to confirm the fabrication of graft polymer as illustrated in Fig 2.

βCD spectra showed characteristics functional groups peaks including C-H stretching at 2925.06 cm^−1^, O-H stretching at 3285.62 cm^−1^, H-O-H bending at 1539.70 cm^−1^, C-O band of COOH at 1149.64 cm^−1^, and C-O-C bending at 1021.43 cm^−1^ [40]. PVP’s FTIR spectrum shows CH stretching characteristics peaked at 2854.23 cm^-1^. The stretching of the carbonyl group (C = O) was noticed as a prominent peak at 1536.35 cm^-1^. The amide band III (C–N stretch) had a significant peak at 1321.01 cm^-1^. The structural peaks in the FTIR spectra of AMPS were 1660.41 cm-1 and 1563.94 cm^-1^, respectively, showing C = O stretching (Amide I band) and N-H bending (Amide II band). The presence of the SO_3_H group in AMPS was confirmed by characteristic peaks at 1236.09 cm^-1^ and 1365.51 cm^-1^, which showed symmetrical and asymmetrical stretching of the S = O group. The spectral peak at 2985.66 cm^-1^ revealed C-H stretching of-CH_2_. The S-O-C group is represented by a prominent absorption band in the range of 1077.85 cm^-1^ to 832.75 cm^-1^ [13–41]. The peaks at 3282, 2858, and 1422.45 cm^-1^ in the FTIR spectra of rosuvastatin correspond to cyclic amines CH stretching, C = O stretching, and O-H bending, respectively [19,20] The infrared spectrum of RST-loaded nanogel revealed cross-linking between the polymer and monomer, as well as the presence of major peaks of CD (polymer), AMPS (monomer), and Rosuvastatin (drug) with minor frequency shifts from 1668.19 cm^-1^ to 1641.96 cm^-1^, and the amide I band has changed marginally. It has also been found that the Amide II band shifts from 1563.94 cm^-1^ to a low frequency of 1487.40 cm^-1^. The peak suggesting S = O group stretching has been changed from 1365.51 cm^-1^ to 1397.60 cm^-1^. The carbonyl stretching in PVP was moved to a high frequency of 1721.08 cm^-1^, with a peak at 1518.35 cm^-1^. CO has modified the intensity of the peak between 1041.49 cm^-1^ and 1397 cm^-1^. The crosslinking between the polymers that leads to the creation of nanogels is confirmed by the OH hydrogen bond in βCD/PVP-co-poly (AMPS). The successful integration of medication and the development of stable cross-linked nanogels were indicated by the FTIR spectrum.

### 4.2. Morphological characterization

**Fig 3:**
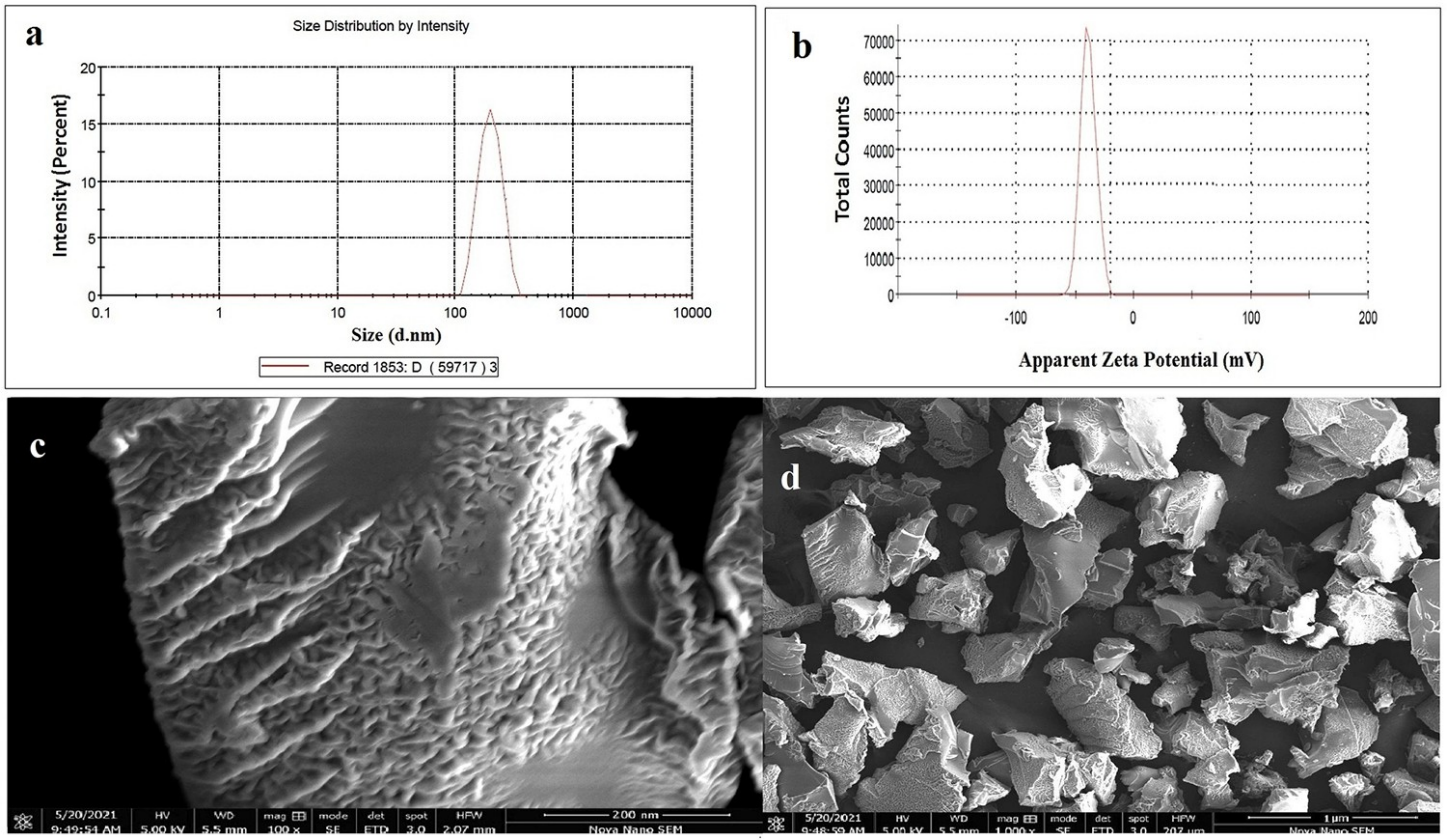
Particle size distribution curve showing low PDI (a) zeta potential curve indicating surface charge for better dispersion (b) and morphological characteristics (c, d) of βCD/PVP-co-poly (AMPS) nanogels.

The shape of a drug delivery system is important for the successful loading and distribution of chemical moieties. A porous and fluffy structure was observed in the pores of the freeze dried nanogels loaded with drug as shown in Fig 3. Due to hydrophilic groups in polymers and monomers, many small pores are present. The nanogel porosity makes them more receptive to water and drugs when they come into contact with solvents. The RST loading was observed as white patches in the porous structure. Lyophilization increased the nanogels’ porosity, it is also possible that nanogels’ uniformity will be affected by the lyophilization process, which is used to remove any remaining or entrapped solvent after drug loading. In a previous study, the porosity of the drug delivery system was also linked to lyophilization [13–42].

### 4.3. PXRD studies

**Fig 4:**
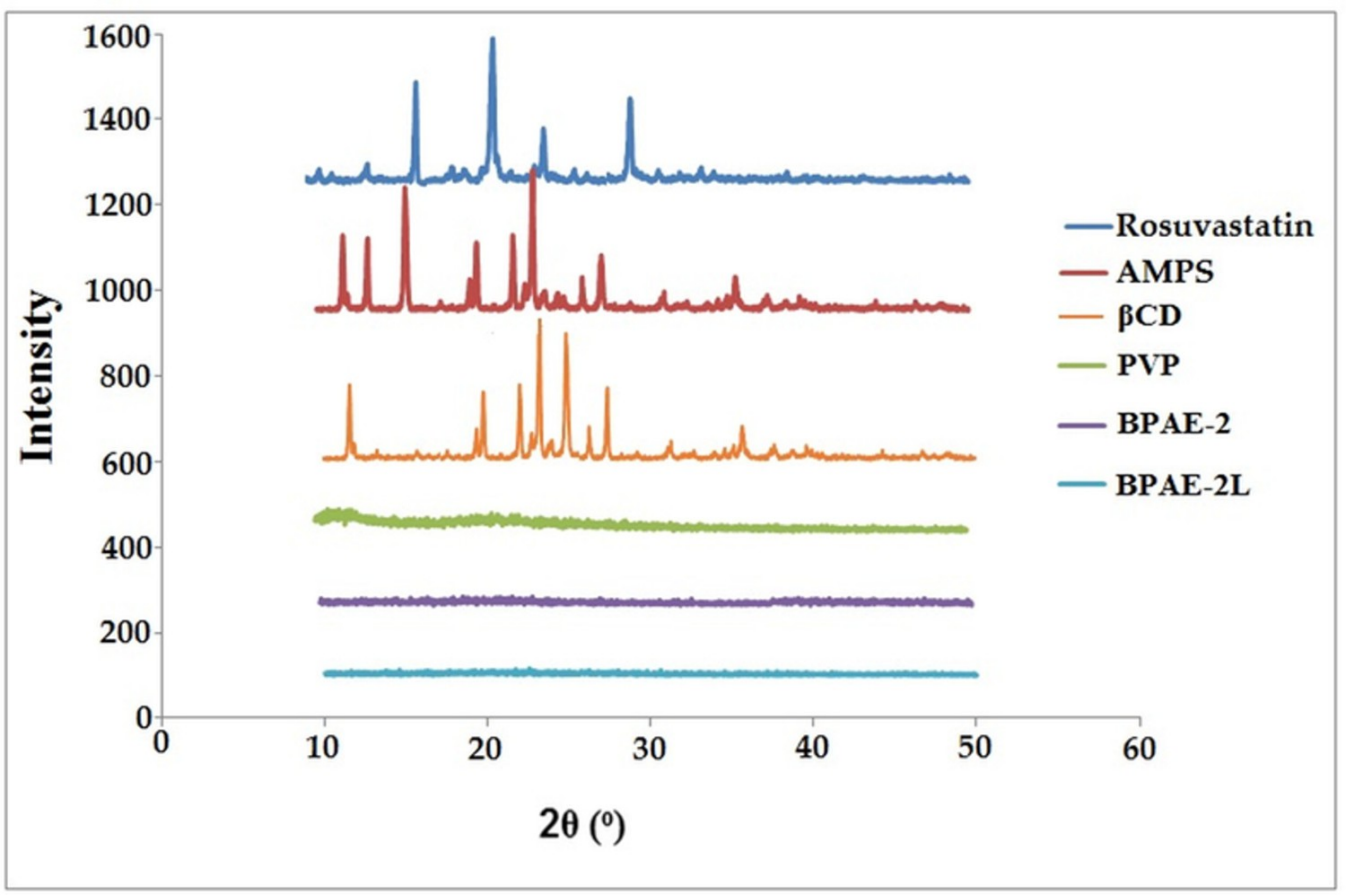
PXRD of Rosuvastatin, βCD, PVP, AMPS, unloaded (PBCD-2) and loaded (PBCD-2L) nanogels showing crystalline nature of components and amorphous nature of nanogels.

XRD is one of the most significant factors to investigate while trying to increase the solubility of a chemical entity that is poorly water soluble. Some chemicals’ polymorphic variations have a major impact on their solubility, dissolution, and bioavailability. To determine if rosuvastatin was crystalline or amorphous, powder X-ray diffractometry was performed. Fig 4 shows the PXRD diffractograms of βCD, PVP, AMPS, RST, unloaded, and drug-loaded nanogels. The XRD pattern of the pure drug (rosuvastatin) showed sharp peaks at 2θ = 15.04^o^, 19.11^o^, 22.45^o^ and 29.3^o^. PXRD examination of βCD showed characteristics peaks at 2 θ = 12.75°, 19.90°, 22.23°, 23.03°, 24.85°,28° and 34.27° [20]. On the other hand, PXRD diffractogram of AMPS clearly showed its characteristics peaks at 2 θ = 11.50°, 12.85°, 15.61°, 19.95°, 21.79°, 23.43°, 26.10° and 27.01° [23]. The absence of the intense peaks in nanogels that were not loaded favoured the system’s amorphous character. This was owing to the polymeric network’s amorphous structure, which attenuated the polymer peak. It was determined that the drug had been successfully encapsulated in nanogels by a decrease in the intensity of the drug peaks. Solubility and dissolution rate can be improved in amorphous systems due to their higher intermolecular energy level, while molecular mobility is increased in amorphous systems [43].

### 4.4. Particle size analysis

An important parameter is the particle sizes analysis of nanogels, because they play a dynamic part in the dissolution rate, solubility, and release of the drug. Ostwald Freundlich’s equation states that solubility is directly linked with particle size, i.e. solubility increases with the reduction of particle size. Nanotechnology is a remarkable approach for poorly soluble medications that have a profound effect on biopharmaceutical performance because smaller particle size promotes both greater solubility and higher-surface bioavailability [44,45].

The feed ratio of fundamental ingredients and the system’s crosslink density has a big impact on particle size. Polymer and monomer concentrations increased from BPAE-1 to BPAE-3 and BPAE-6 to BPAE-9, whereas cross-linker concentrations increased from BPAE-4 to BPAE-5 (Table 1). Particle sizes of unloaded nanogels rise from BPAE-1 to BPAE-3 and BPAE-6 to BPAE-9, while decreasing from BPAE-4 to BPAE-5, as shown in Table 2.

**Table 2:** Average particle size of unloaded and loaded nanogels.

Formulation code	Average particle size of unloaded nanogels	Average particle size of loaded nanogels
BPAE-1	173± 2.34	201±2.64
BPAE-2	178±3.41	207±3.01
BPAE-3	182±2.71	211±2.97
BPAE-4	162±2.36	191±2.65
BPAE-5	158±4.61	187±2.31
BPAE-6	184±1.82	213±3.95
BPAE-7	189±2.03	217±1.91
BPAE-8	185±2.37	216±3.41
BPAE-9	190±2.83	221±0.89

Note. All values are expressed as mean ± SD (n = 3).

**Fig 5:**
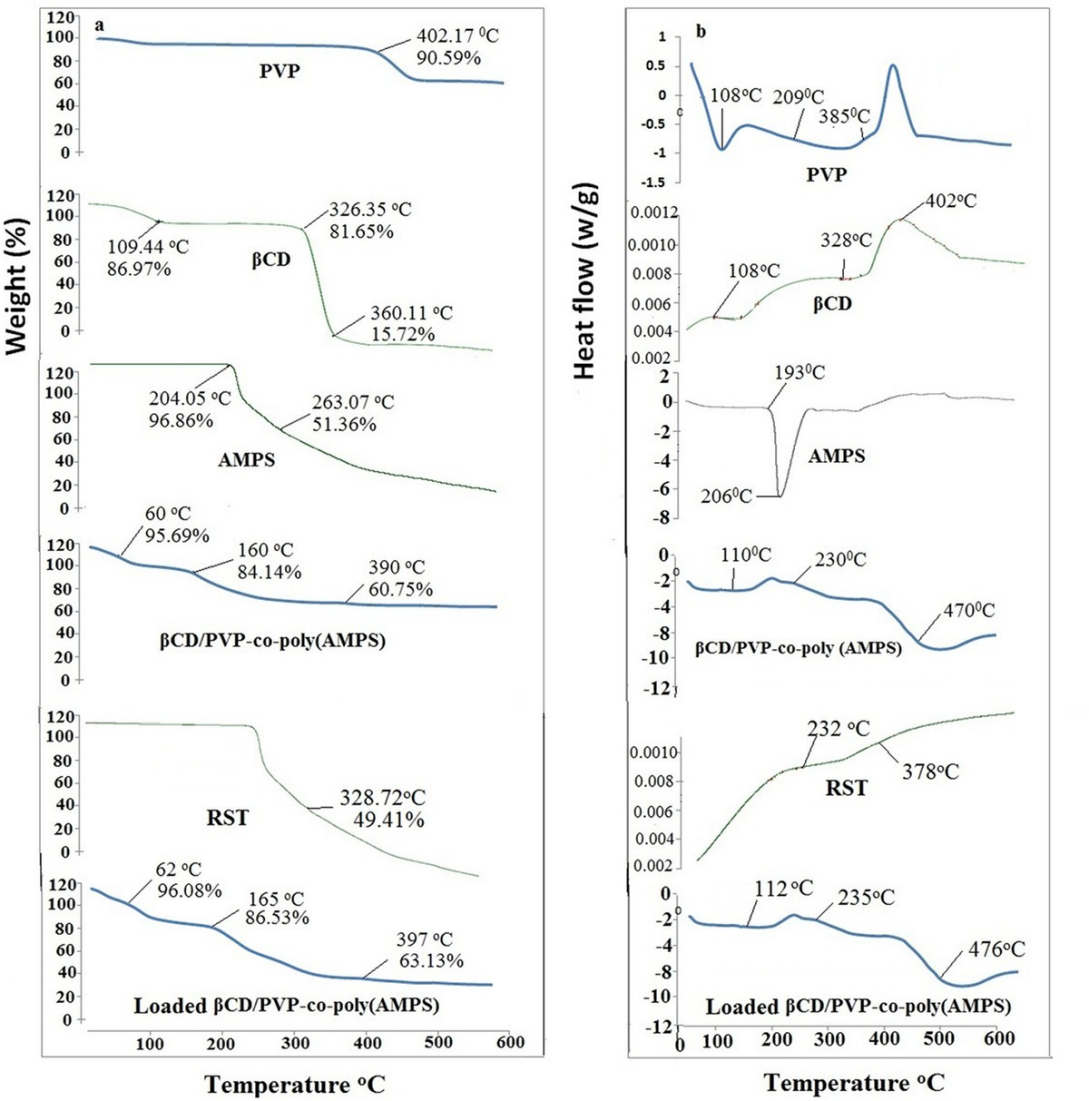
TGA thermogram (a) and DSC curve (b) of βCD, PVP, AMPS, RST, unloaded and RST loaded βCD/PVP-co-poly (AMPS) nanogels indicating better thermal stability of nanogels than individual components.

The increase in particle size with increased polymer and monomer concentrations may be connected to nanogels hydrophilicity and crosslink density. Decreased nanoparticle sizes were also observed with increasing crosslinker concentration, due to the increased crosslink density, which resulted in smaller particles. Another study found that increasing the concentration of crosslinker resulted in particle size reductions [46]. It was also found that RST-loaded nanogels were larger than unloaded nanogels (29 nm), indicating successful drug loading. A hydrophobic cavity exists within the βCD molecule, which explains the increase in particle size. Previously, a drug delivery system’s particle size increased after being loaded with a hydrophobic drug [47]. Many researchers have studied the effect of particle size on dissolution, solubility and bioavailability [48]. Because of the increasing surface area and narrowing of the diffusion area, saturation solubility increases as particle size decreases. All of these variables work together to boost solubility, which boosts bioavailability [49]. As demonstrated in Table 2 and depicted in Fig 3, the particle size of nanogels (BPAE-2) was in the range of 178 ± 3.41 d.nm with the value of polydispersity index (PDI) of 0.345. Similarly, value of zeta potential was found to be in the range of -37 ± 2.45 mV as illustrated in Fig 5. Drug loaded nanogel with optimized particle size were found to be in the range of 207± 3.01. The solubility of nifedipine was successfully increased in a previous study using nanocrystals [50]. A previous study reported that nanogels with reduced particle size were employed to improve the solubility of lipophilic drug olanzapine [6].

### 4.5. Micromeritics studies

The micromeritics analysis is necessary to calculate to identify the flow properties for particulate material. The good flow properties are confirmed if the angle of repose is less than 30° and Carr’s index lies between 13% to 19%. Whereas, the Hausner’s ratio should be less than 1.25 [51,52]. Materials with poor flow characteristics are difficult to use leading to inadequate stacking of medication therefore it is essential to calculate the flow properties of fabricated nanogels. The obtained results of rheological parameters are given in Table 3. All the results were found between the pharmacopoeial limits and indicate the fabricated samples of nanogel have good flow properties [24,25].

**Table 3:** Rheological parameters of fabricated formulations (BPAE-1 to BPAE-9).

Formulation	Bulk density (g/ml)	Tapped density (g/ml)	Angle of repose	Hausner’s ratio	Carr’s Index (%)
**BPAE-1**	0.637	0.739	22.30	1.160	0.138
**BPAE-2**	0.656	0.749	22.40	1.141	0.124
**BPAE-3**	0.698	0.787	23.40	1.127	0.113
**BPAE-4**	0.631	0.723	23.80	1.145	0.127
**BPAE-5**	0.626	0.742	22.90	1.185	0.156
**BPAE-6**	0.673	0.763	25.60	1.133	0.117
**BPAE-7**	0.669	0.756	23.50	1.130	0.115
**BPAE-8**	0.656	0.709	22.50	1.111	0.100
**BPAE-9**	0.673	0.760	22.60	1.129	0.114

### 4.6. Thermal investigation

Thermogravimetric analysis (TGA) and differential calorimetric scanning (DSC) analysis were performed to assess the thermal stability of the developed nanogels, the mass loss, and thermal transitions associated with evaporation (endothermic) or decomposition (exothermic) events. Nanogels made from βCD/PVP-co-poly (AMPS) were tested using TGA and DSC as shown in Fig 5(A) and 5(B) respectively.

The TGA thermogram of pure βCD shows three prominent decomposition phases, with the first one occurring at 109.44°C, i.e. 14.03 percent having (curve mass change) Δm = 86.97 percent due to the loss of bound water. At 326.35°C, a second significant weight loss of 19.35 percent (Δm = 81.65 percent) was found, indicating degradation in the side chain and branches of the polymer, leaving the main chain to degrade at a higher temperature of 360.11°C (Δm = 15.72 percent) [26]. The TGA thermogram of pure PVP shows high thermal stability, with decomposition beginning at 402.17°C (Δm = 90.59%) and only 38.21 percent weight loss up to 480°C (Δm = 61.89%). In the thermogravimetric curve, AMPS showed two separate degradation phases. The loss of bound water is responsible for the first breakdown phase at 204.05°C (Δm = 96.86%). Another breakdown phase of AMPS is related to combustion above the melting point at 263.07°C (Δm = 51.36 percent) [23].

βCD/PVP-co-poly (AMPS) unloaded optimized formulation (BPAE-3) exhibits three distinct degradation phases. Initial decay in TGA thermogram was observed at 60°C (Δm = 95.69%) due to the loss of bound water. The sulphonic acid functional group attached to the polymeric backbone decomposes at 160°C (Δm = 84.14%) leaving the main chain, which degrades further at 390°C (Δm = 60.75%). 40% of the mass remained at 390°C in the thermogram of the fabricated nanogel formulation. Crosslinking between the polymers and the monomer is responsible correspond to good thermal stability. TGA curve of pure RST exhibited 49.41% of mass loss at 328.72°C. Besides, βCD/PVP-co-poly (AMPS) loaded nanogel also three distinct degradation zone phases first at 62°C (Δm = 96.08%) second at 165°C (Δm = 86.53%)and the last was at 397°C (Δm = 63.13%) representing good thermal stability after drug is loaded [20].

According to Fig 5(B), DSC was performed on purified βCD, PVP, and AMPS, and on loaded optimized formulation (BPAE-3L) made from βCD/PVP-co-poly (AMPS). The first endothermic peak in the DSC curve of βCD occurs at 108°C, which is associated with the removal of bound water from the sample. Another peak was viewed at 328°C on the DSC thermogram, but it’s only a minor peak. Thermal decomposition can be seen as an endothermic peak at 381°C [26]. PVP’s DSC curve shows an endothermic peak at 108°C corresponding to the glass transition temperature of the material. This temperature indicates how much bound water has been removed. Due to an unspecific solid-solid transition, the DSC thermogram of PVP displays a tiny peak at 209°C. Furthermore, two peaks at 108°C and 209°C were viewed but at 385°C a deep endothermic peak was visible in the PVP sample exhibiting thermal degradation [27]. The melting point of AMPS is represented by the first endothermic peak at 193°C on the DSC curve. Decomposition is indicated by an endothermic peak at 206.61°C [23]. DSC curve of unloaded nanogels shows melting of formulation at 110°C. It also has an endothermic peak at about 230°C, which could be their melting or oxidative degradation point. Major degradation of βCD/PVP-co-poly (AMPS) nanogels observed at 470°C. Due to increased crosslinking density and branching, in pure PVP, the endothermic peak at 390°C is shifted to 470°C. A characteristic endothermic peak was observed in DSC curve of pure RST at 232°C while another peak was observed at 378°C on final combustion. DSC curve of RST loaded nanogels shows melting of formulation at 112°C. It also has an endothermic peak at about 235°C, which could be their melting or oxidative degradation point. While major degradation of βCD/PVP-co-poly (AMPS) nanogels formulation observed at 476°C [20].

### 4.7. Sol-gel analysis

It is possible that some of the polymer and monomer components of the reaction will remain unreacted during the free radical polymerization process. Polymer and monomer crosslinking and uncrosslinking estimated by using sol-gel fraction analysis. In the synthesis of nanogels, the sol fraction is the amount of uncrosslinked polymer and monomer that has not been crosslinked. During the Soxhelt extraction process, unreacted polymer and monomer are solubilized and their extent is determined by the sol fraction. The gel is the insoluble fraction that exhibits the fraction of crosslinked polymer and monomer. The crosslinking reaction between the constituents will be more satisfactory if the gel fraction is higher. Polymers, monomers, and crosslinker were used in varying proportions and their effects on the sol-gel fraction were observed (Table 4).

**Table 4:** Drug loading and drug loading percentage, Sol-Gel fraction and % water content of all βCD/PVP-co-poly (AMPS) nanogels.

Sr.#	Formulation Code	Amount of Drug Loaded (mg)	% Drug Loading	Sol fraction (%)	Gel fraction (%)	% water absorption
**1**	BPAE-1	30.45±3.26	76.13±0.21	11.39±1.112	88.61±2.5392	12.65
**2**	BPAE -2	33.01±4.83	82.53±0.16	7.86±3.117	92.14±2.567	13.23
**3**	BPAE -3	35.58±8.06	88.95±0.33	4.81±1.053	95.19±1.754	14.44
**4**	BPAE -4	30.25±4.12	75.63±0.09	10.43±4.223	89.57±3.520	11.98
**5**	BPAE -5	27.14±5.55	67.85±1.01	3.95±2.0485	96.05±1.780	11.54
**6**	BPAE -6	31.80±3.71	79.51±0.81	9.68±4.534	90.32±2.840	13.76
**7**	BPAE -7	34.75±2.13	84.38±0.06	6.06±2.589	93.94±3.21	13.98
**8**	BPAE-8	31.97±6.14	79.93±0.11	9.68±1.936	90.32±4.40	13.73
**9**	BPAE-9	34.00±2.01	85.04±0.08	5.06±2.495	94.94±2.225	14.01

Note. All values are expressed as mean ± SD (n = 3).

Sol-gel analysis revealed that when the polymer concentration increased, the gel fraction increased. (βCD, PVP) and monomer concentration (AMPS). Free radicals (active sites) for free-radical polymerization increased as polymer and monomer concentrations were increased in the formulation, resulting in a higher gel fraction. Kaleem Ullah and Yin Lichen *et al*. also found that the gel fraction increased as polymer and monomer (AMPS) concentrations increased [53,54]. Crossliner (MBA) also has an effect on sol gel fraction, which we examine in our study. Increased gel fraction with increased crosslinker concentration owing to the reason that during the polymerization phase, as the MBA concentration increased, it supplied more binding sites. A previous study found that an increase in MBA content results in the generation of more free sites for the polymerization reaction to complete [55,56]. By selecting appropriate concentrations of reactants, it is possible to carry out a reaction properly.

### 4.8. Solubility studies

**Fig 6:**
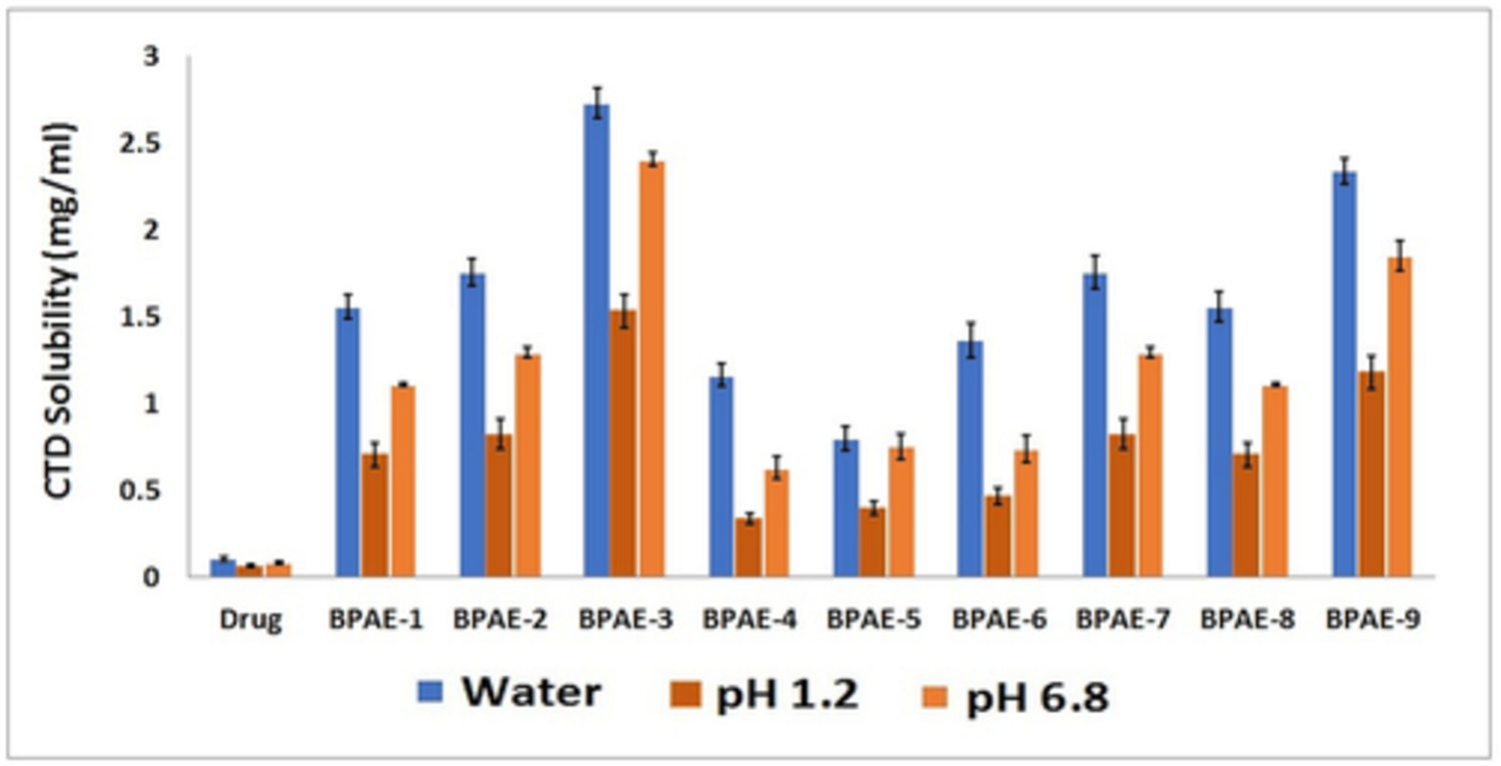
Comparative solubilization (%) of Rosuvastatin in pure form and developed βCD/PVP-co-poly (AMPS) nanogels in distilled water (highest solubility), phosphate buffer of pH 6.8 (high solubility) and HCl buffer of pH 1.2 (low solubility).

The drug and nanogels’ solubility studies were carried out in buffers with pH values of 1.2 and 6.8 as well as in water. Considering that Rosuvastatin is an acidic drug, its ionization in higher pH (pH 6.8 and water) will be greater than in lower pH environments (pH 1.2). Solubility outlines of nanogels and the drug alone differed significantly, with nanogels showing excellent solubility. The solubility of the drug was increased with that of nanogels as illustrated in Fig 6. Formulation BPAE -3 prepared with maximum content of the monomer (AMPS) demonstrated 26 folds increase in the solubility profile. Nanogels’ solubility was found to be higher in water than at pH 1.2 and 6.8. There is a possibility that this difference is related to the pKa of the drug. Since the drug’s pKa value is 4.6, its ionization in an aqueous medium will be higher than in pH 1.2 and 6.8 media. pH 6.8 also had a higher solubility profile for nanogels as compared to pH 1.2. In light of βCD’s structural characteristics, it is possible to explain how a solubility enhancement mechanism operates. In contrast, the βCD’s external surface is hydrophilic, while its internal cavity is lipophilic. When a polymer is placed into a βCD based drug delivery system, the internal lipophilic cavity of the polymer suppresses drug hydrophobic features, while hydrophilic functionalities are exposed to the external environment. When a drug’s hydrophilicity is increased, the maximum amount of drug is dissolved in the medium. The increase in RST solubility and dissolution rate may be due to the smaller particle size (increased surface area), wettability, and solubilizing effect of polymers, as well as the amorphous structure of nanogels [57].

### 4.9. Swelling studies

**Fig 7:**
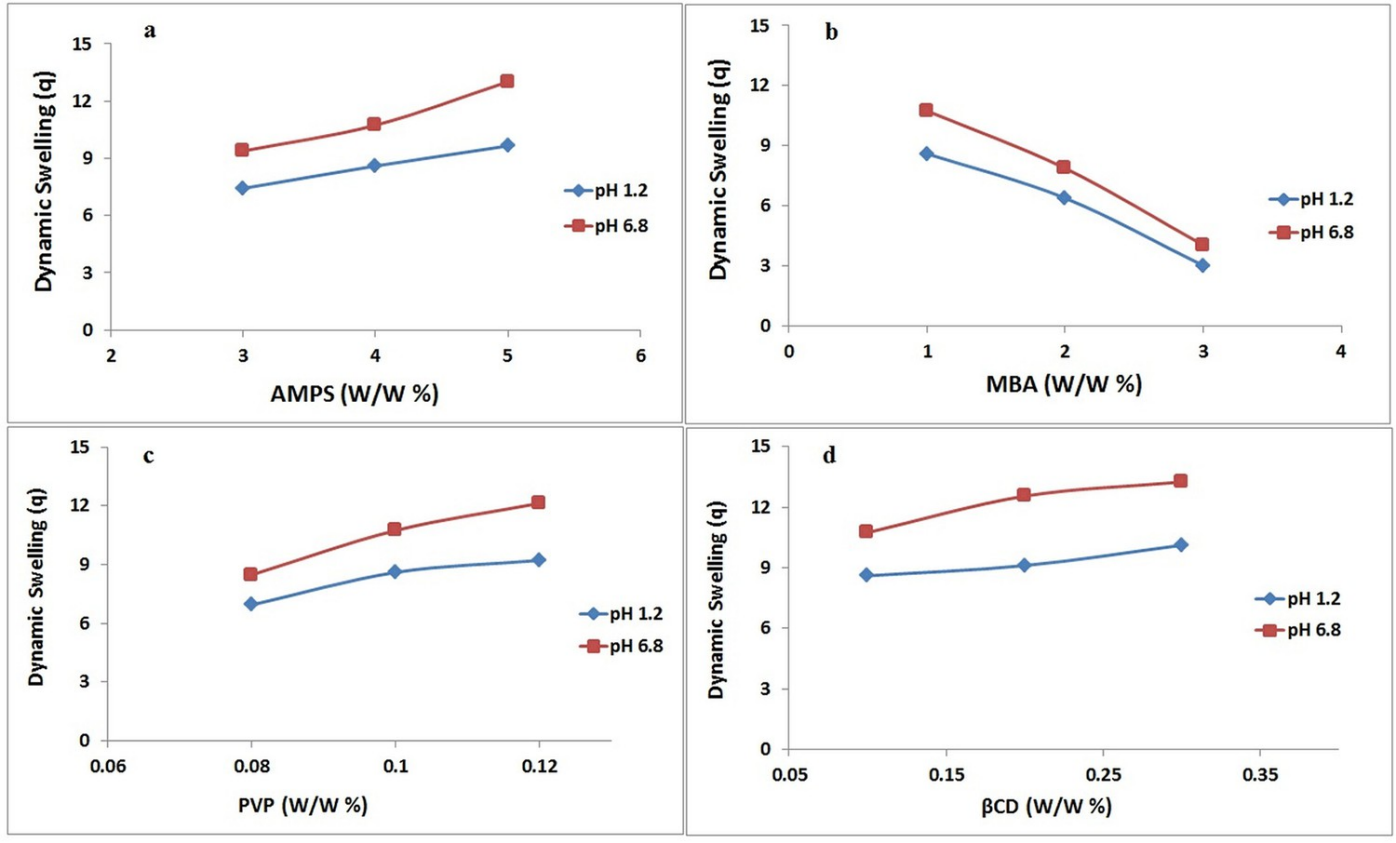
Effect of different concentrations of AMPS (a) MBA (b) PVP (c) and βCD on swelling index at pH 1.2 and pH 6.8 indicating all components have an incremental effect on swelling except MBA having the opposite effect.

Swelling of the drug delivery system had an enormous and direct impact on the properties of the release of drugs and therefore swelling of the developed drug carrier system, i.e. nanogels was important to examine [58]. It demonstrates the permeability and water-carrying capability of nanogels. The influence of pH medium, polymer (βCD, PVP), monomer (AMPS), and crosslinker (MBA) concentration on the swelling properties of produced nanogels was investigated in both acidic (1.2) and basic (6.8) pH mediums [59]. Fig 7 shows the swelling index of all βCD/PVP-co-poly (AMPS) based nanogels at acidic and basic pH. Maximum swelling of nanogels was observed in both pH mediums in 5 to 30 minutes, based on results obtained during the swelling experiment. This was due to the hydrophilic nature of both polymer (βCD, PVP) and monomer (AMPS). Second, Nanogels are nanosized and spongy materials that impart increased swelling capabilities and, as confirmed by the dissolution experiment, facilitate the rapid release of drugs in the aqueous medium [60].

Because the sulfonate anions (-SO_3_) in the monomer (AMPS) were protonated into–SO_3_H, additional crosslinking occurred, resulting in lower swelling ratios in acidic medium, whereas higher pH levels resulted in increased swelling ratios due to strong electrostatic interactions among the SO_3_ groups as a result of ionization [60,61].

To investigate the impacts of polymeric and monomeric concentrations on swelling, βCD, PVP, AMPS, and MBA were utilized in varying amounts during the nanogels’ manufacture. When the polymer concentration is raised, nanogels swell even more. This could be attributed to the crosslinked network’s huge number of hydrophilic moieties. The diffusion of the swelling medium inside the polymeric network increased as the number of hydrophilic moieties rose, resulting in a bigger swelling of the system. Furthermore, increasing the polymer concentration yielded a significant number of functional units for grafting AMPS. All of these factors contributed to an increase in swelling as the polymer content was raised. According to a prior study, increasing the polymer content caused the system to swell [21].

As the concentration of AMPS was increased, the swelling behaviour of nanogels changed dramatically. Nanogels swelled more as AMPS concentration was increased. Nanogels’ swelling properties may be related to the fact that AMPS contain sulfur-containing groups that increase in concentration as the monomer’s concentration increases. This provides a large number of sulphonis groups to be ionized or alternatively, results in maximum electrostatic repulsion. The concentration of AMPS was increased to increase swelling because the hydrophilicity of the crosslinked structure increased as AMPS content was increased [23].

Nanogels expanded less as MBA concentration increased. Increasing the cross-linker concentration reduces nanogel swelling by generating a denser crosslinked network, which prevents considerable swelling. Increased crosslinker concentration resulted in more crosslinked sites in the polymeric network. As a result, the pore diameters of the network shrank. This decrease in pore size resulted in delayed diffusion of swelling medium into the polymeric network of nanogels, according to the findings. When the cross-linker concentration is raised, the nanogels swell less. Sing et al. discovered that when the concentration of MBA grew, the swelling of nanogel decreased [62].

### 4.10. Percentage water absorption

A water absorption study was conducted among fabricated nanogel formulations having different monomer, polymer, and cross-linker. The results are given in Table 4. The high pore volume of the formulation leads to high water absorption. The extent of porosity of the nanogels directly enhanced with the decrease in the cross-linking degree. More the concentration of polymer more will be the number of pores that hold more water. When the polymer concentration is raised, nanogels swell even more and absorb more water. This could be attributed to the crosslinked network’s huge number of hydrophilic moieties. The diffusion of the swelling medium inside the polymeric network increased as the number of hydrophilic moieties rose, resulting in a high percentage of water absorption. An increase in crosslinker content results in increased crosslinking and decreased pore volume. This results in a decreased percentage of water absorption resulting decreased swelling. BPAE -5 gave minimum water absorption as the concentration of MBA is increased which give rise to decreased swelling. Nanda et al. 2013 and Sadeghi et al. reported similar results during their study that by changing the amount of crosslinking agent, polymer and monomer change in water absorption content is observed [31–63]

### 4.11. Drug loading percentage

Nanogels were loaded using the post-loading method and results are shown in Table 4. Nanogels exhibited a drug loading rate greater than 70%. The formulation BPAE -3 showed the best results, with a percent drug loading of nearly 90 percent. The factor of increasing crosslinker concentration may be linked to the minimum loading of formulations BPAE -5 and BPAE -6. As the cross-linker concentration was increased, a dense, cross-linked network was formed. Solvent diffusion into such a dense, cross-linked network is difficult. Furthermore, swelling of the system, which is important for loading and release of drugs, was reduced. All of these factors combined to limit the amount of drug that could be loaded into BPAE -5 and BPAE -6 [64].

### 4.12. *In vitro* drug release studies

**Fig 8:**
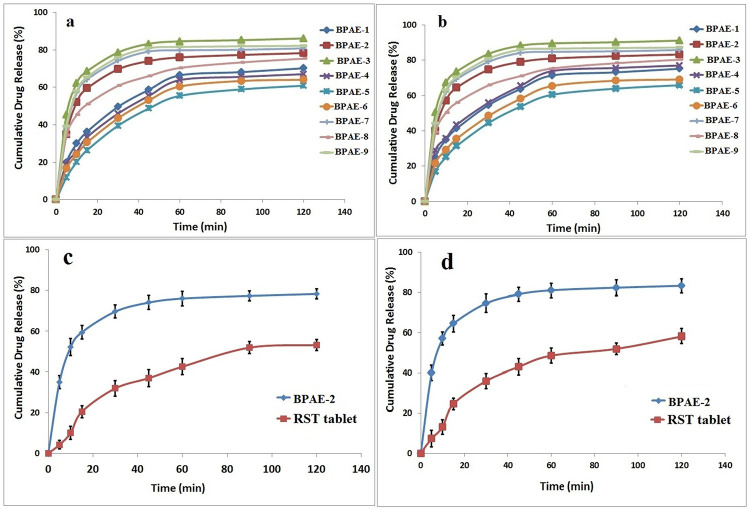
The drug release profile of fabricated nanogels at pH 1.2 (a), pH 6.8 (b) and of commercially available tablets at pH 1.2 (c) and pH 6.8 (d) indicating better release profile of nanogels.

A dissolution study was carried out in phosphate buffers of pH 6.8 and HCl buffer of pH 1.2 to determine the i*n vitro* drug (RST) release from βCD based nanogels’ drug delivery system. A study was conducted with the aim of comparing drug release with nanogels for all developed nanogel formulations as well as commercially available RST tablets. Rosuvastatin is acidic drug having a pKa value of 4.6, its dissolution favors under basic condition (pH 6.8). It was seen in the study, that the maximum drug was released in 5 to 30 minutes from all formulations of developed nanogels in both acidic and basic pH as illustrated in the Fig 8. This is because the system (nanogels) is hydrophilic and amorphous, which promotes swelling and the release of drugs in higher pH, also confirmation of the swelling experiment. Second, protonated sulfonate anions (–SO_3_^–^) in monomers (AMPS) caused additional cross-linkages, resulting in a reduction in the swelling proportion in acid medium while in the case of higher pH due to significant electrostatic repulsion between–SO_3_^–^ groups as a result of ionisation of acidic medium. The sulfonate groups have a strong interaction. When developed with hydrophilic polymers and crosslinked with water-loving monomers, nanogels form a highly water interacting network that swells abruptly with access to both pH 1.2 and 6.8 aqueous medium, and the drug tugging in the structure of the gel bursts out into the aqueous medium. As a result, drug particle solubility is improved as a result of their unique interaction with the medium. From a different perspective, the solubility improvement mechanism of these systems, as well as the great drug release, could be explained by the increased swelling, better solubility, and superior drug release as mentioned above [60].

Drug dissolution may be enhanced by the polymer’s high swelling ability and hydrophilic properties. Because nanogels are hydrophilic, they can absorb large amounts of solvent and the polymer dissolves much more quickly than the drug. The drug will dissolve more quickly because there will be reduced interfacial tension between the drug and the water since this activity can act on the hydrodynamic layer around the nanogels. The ground technique, like the above, may speed up rosuvastatin dissolving by increasing the total surface area of the drug’s interaction with βCD or decreasing the crystallinity of rosuvastatin [65]. The dissolution studies established that βCD enhances the dissolution profile of a drug. The amount of medication released was significant, and the outcomes were satisfactory. Formulations that have fewer release of drug, BPAE-4 and BPAE-5 were restricted. Because of the increased content of MBA, these two formulations may have a limited drug release due to a dense and tight crosslinked network. It was found that as the crosslinking density increased, the swelling and diffusion of dissolution medium into the crosslinked network were affected, resulting in limited drug diffusion from the polymeric network [66]. On the other hand, the in vitro drug release profiles of commercially available Rosuvastatin tablets Rovista were compared to those of nanogels. All dissolution media showed maximum drug release, indicating that the drug’s solubility had been greatly enhanced, whereas the drug’s release from commercially available tablets was less and delayed, indicating its low solubility as compared to developed nanogels. The drug was released slowly in aqueous pH 1.2 and pH 6.8 medium, with maximum release happening after one hour. The nanogel created were able to reach maximum drug release in 30 minutes due to their fast release.

### 4.13. Toxicological studies

In our study, the toxicity of the polymeric nanogel system was also evaluated through hematological, biochemical, and histopathological parameters. Hematological analysis was performed to assess the effect of βCD-based polymeric nanogels on the biological system. Blood samples of test animals were collected immediately in ethylene diamine tetra acetic acid tubes (EDTA) to prevent blood coagulation and then various parameters were analyzed related to biochemical analysis of blood [54]. Two groups of total 12 rabbits have been arranged in two groups with each having 6 rabbits; Group I (control) and Group II (Treated with nanogels formulation). They are carefully placed in separate cages. Various parameters (body weight, food and water intake, dermal & ocular toxicity and mortality) were evaluated as reported in Table 5.

**Table 5:** Clinical observations of an acute oral toxicity study for βCD/PVP-co-poly (AMPS) nanogels.

Observations	Group I (control)	Group II (treated with nanogels)	p-value
**Sign of illness**	Nil	Nil	
**Food intake (g)**			
Preliminary to therapy	67.37 ± 1.62	68.42 ± 2.19	0.631
Day 1	69.12 ± 1.52	71.21 ± 3.17	
Day 7	73.14 ± 1.03	73.29 ± 2.51	
Day 14	75.42 ± 2.08	75.61 ± 4.03	
**Water intake (ml)**			
Preliminary to therapy	189.14 ± 4.19	187.14 ± 2.11	0.521
Day 1	191.63 ± 2.33	190.62 ± 1.62	
Day 7	201.01 ± 1.42	196.19 ± 1.02	
Day 14	203.21 ± 3.19	199.64 ± 3.41	
**Body weight (kg)**			
Preliminary to therapy	2.03 ± 0.01	2.08 ± 0.05	0.417
Day 1	2.06 ± 0.03	2.11 ± 0.01	
Day 7	2.07 ± 0.07	2.12 ± 0.04	
Day 14	2.09 ± 0.06	2.14 ± 0.03	
**Other Toxicological Observations**			
Ocular toxicity	Nil	Nil	
Dermal toxicity	Nil	Nil	
Simple irritation	Nil	Nil	
Mortality rate	Nil	Nil	

Note. All values are expressed as mean ± SD (n = 12).

The value of “p” less than 0.05 was considered significant.

In our study, hematological, biochemical and histopathological parameters have also been used to assess the toxicity of polymeric nanogel systems. Blood analysis findings have confirmed that the developed nanogels are biocompatible, since the results are within normal ranges as reported in Table 6.

**Table 6:** Hematology, biochemical parameters and consequences of orally administered βCD /PVP-co-poly (AMPS) nanogels on organ weight (g) of rabbits.

**Biochemical blood analysis**			
**Parameter/test**	**Group I (control)**	**Group II (treated with nanogel)**	**p-value**
White blood cells ×10^9^/L	6.93 ± 0.81	7.21 ± 0.24	0.230
Platelets × 10^9^/L	4.23 ± 1.26	4.61± 1.19	0.851
Hemoglobin (g/dl)	12.96 ± 1.39	13.06 ± 1.36	0.501
Red blood cells 10^6^/mm3	5.97 ± 0.76	6.15 ± 0.21	0.744
Neutrophils (%)	55.04 ± 2.62	54.95 ± 3.01	0.327
Lymphocytes (%)	65.10 ± 0.08	64.15 ± 1.32	0.901
Monocytes (%)	3.51 ± 2.04	3.52 ± 1.09	0.567
Mean corpuscular Volume MCV(%)	63.72 ± 1.01	66.08 ± 1.42	0.366
Mean corpuscular hemoglobin MCH (pg/cell)	24.17 ± 1.81	27.47 ± 1.41	0.793
Mean corpuscular hemoglobin concentration MCHC (%)	31.07 ± 1.26	32.53 ± 1.21	0.250
**Biochemical parameters**			
ALT/SGPT (IU/L)	29.10 ± 1.41	30.21 ± 1.32	0.606
AST/SGOT (IU/L)	142.0 ± 0.17	141.17 ± 0.51	0.670
Cholesterol (mg/dl)	62.42 ± 3.14	65.16 ± 2.15	0.935
Creatinine (mg/dl)	0.94 ± 0.02	0.96 ± 0.08	0.914
Serum urea (mg/dl)	14.23 ± 0.04	16.70 ± 0.03	0.885
Triglycerides (mg/dl)	53.37 ± 2.47	54.27 ± 2.18	0.873
Serum uric acid (mg/dl)	3.24 ± 0.13	3.39 ± 0.19	0.741
**Organ weight(g) of Rabbits**			
Heart	4.09 ± 0.49	3.39 ± 0.58	0.468
Liver	10.08± 1.09	9.23 ± 1.22	0.704
Stomach	13.29 ± 0.27	11.61 ± 0.36	0.931
Kidney	10.83± 1.29	12.56 ± 1.01	0.879
Lung	9.53 ± 0.24	8.91 ± 0.42	0.369
Spleen	1.32 ± 0.11	1.39 ± 0.18	0.960

Note. All values are expressed as mean ± *SD* (*n* = 12).

The value of “p” less than 0.05 was considered significant.

**Fig 9:**
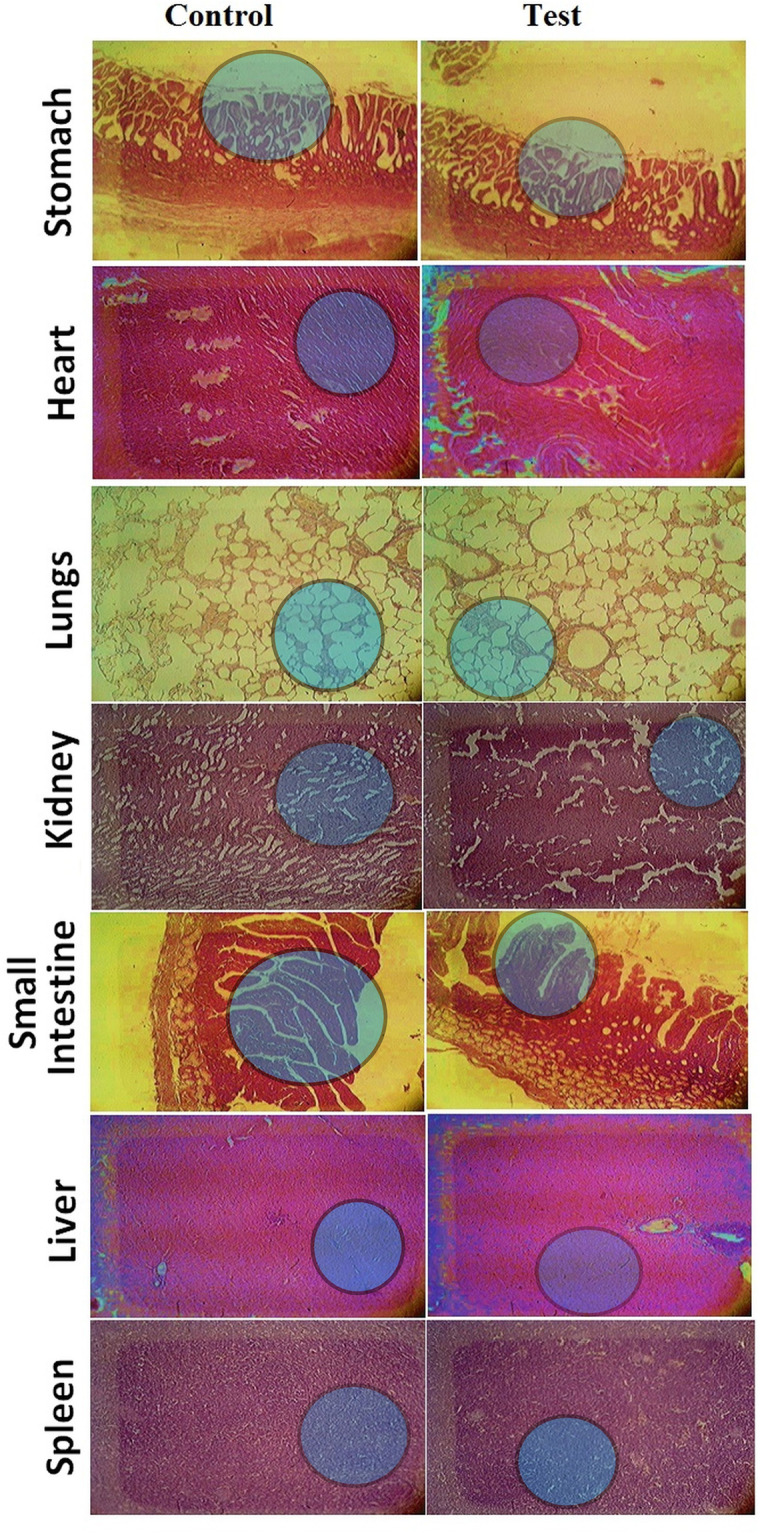
Histopathological tissues examination of different organs from both control and nanogel-treated group indicating no major difference.

Also, histopathological tests to detect the toxicity of the nanogels in vital organs were carried out. To this aim, the test group animals were sacrificed on the fourteenth day, removing and weighing down vital organisms such as the heart, kidneys, stomach and lungs, bowel, and spleen. All vital organs have been individually removed and immersed in a 10% formalin solution. For histological tissue testing using an optical microscope, several tissue slides were made. Gross microscopic examinations of vital bodies showed no signs of the lesion, inflammatory infiltration, disturbance, deformation, hyperemia, and pathological variations of all types within vital bodies as illustrated in Fig 9.

### 4.14. Hemolytic activity

According to the standards given by ASTM for assessment of hemolytic characteristics of materials the nanogel was found to be hemocompatible. The obtained results are given in Table 7.

**Table 7:** Blood haemolysis index after treatment with nanogel samples for 3 h at 37°C.

Sample code	Sample	Percentage Haemolytic Index
A	Nanogel (0.1 mg/ml)	1.399 ± 0.567
B	Nanogel (0.3 mg/ml)	2.023 ± 0.331
C	Nanogel (0.5 mg/ml)	2.987 ± 0.654
C+	Positive control (d.w)	98.643 ± 0.342
C-	Negative control (d.w)	0

* A (Nanogel 0.1 mg/ml), * B (Nanogel 0.3 mg/ml), * C (Nanogel 0.5 mg/ml), * C+ (Positive control), * C- (Negative control).

The optimized formulation BPAE-3 was selected for the hemolytic study. The release of hemoglobin from RBCs indicates that the fabricated nanogel is completely non-hemolytic at tested concentration although the hemolytic index may increase by enhancing the concentration of the gel. Q Song et al. also reported the non-hemolytic outcomes of cyclodextrane based nanogel during their study at nanogel responsiveness on tumor microenvironment [38].

### 4.15. *In vivo* pharmacokinetics studies

Before application of HPLC method to the pharmacokinetic analysis, its validation was done. For intraassay and interassay, precision accuracy was determined and CV% was found to be within acceptable range (0.7–5.7%,). The lower limit of quantification was found to be 1 ng/ml and recovery of sample was up to 95%. Plasma level estimated by calculating the peak areas was plotted against time. Analysis of different pharmacokinetic parameters of RST was made after administration of drug loaded nanogels and commercially available RST tablets to the experimental animals as shown in Table 8.

**Table 8:** Mean values of pharmacokinetic parameters of rosuvastatin following administration of oral tablet and oral nanogel in rabbits (*n* = 12).

Pharmacokinetic parameter	Commercial Tablet	Hydrogel Nanoparticles	p-value
*C*_max_ (ng ml^-1^)	41.11 ± 2.07	83.34.34 ± 8.23	0.011
*T*_max_ (h)	4 ± 0.72	3 ± 0.17	0.135
t_1/2_ (h)	7.21 ± 3.41	8.60 ± 9.87	0.464
AUC _0–t_ (ng ml^-1^.h)	514.43± 39.58	923.44± 69.37	0.0001
AUC _0–∞_ (ng ml^-1^.h)	562.80± 27.67	1061.56 ± 109.43	0.0008
AUMC (ng/ml*h^2)	6750.24	13967.97	0.004
MRT (h)	11.99 ± 1.94	13.16 ± 13.49	0.017

Note. All values are expressed as mean ± *SD* (*n* = 12).

The value of “p” less than 0.05 was considered significant.

**Fig 10:**
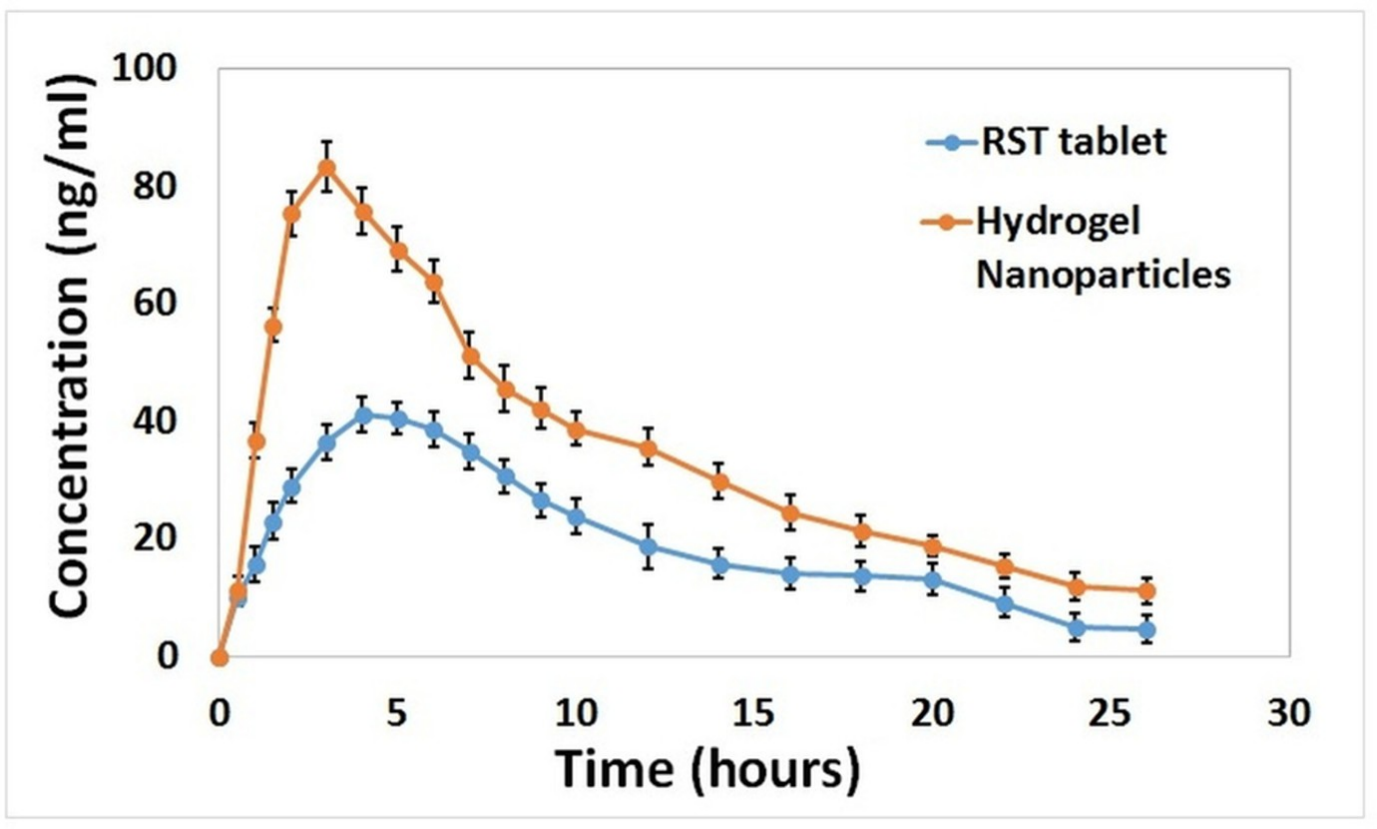
Rabbit plasma concentrations of rosuvastatin HCl after oral administration of rosuvastatin commercially available tablets and loaded nanogels showing better bioavailability of nanogels.

Values of Cmax for nanogel and RST tablets were approximately 83 and 41 ng/ml, respectively as illustrated in Fig 10. A rapid drug release pattern was observed in the case of nanogels as compared to RST Tablets that favor enhanced bioavailability. In a similar way higher AUC_0–t_ of prepared nanogels proved that more drug release from developed formulations might be due to complex formation with βCD and enhanced solubility of the drug in the gastric medium. Results of AUC _0–t_, AUC _0–∞_ and AUMC were higher and significant (p < 0.05) fo nanogel formulation. The half-life (t_½_) of commercially available RST tablets is 7 hr as compared to nanogels for which it is 8 hr. It is concluded from the above results that the solubility and bioavailability of drug RST were successfully improved when it is loaded in nanogels due to the complex formation of the drug with a carrier system [39].

### 4.16 Stability studies

Results of stability analysis proved the stability of developed gel during study period. Insignificant variations were detected in physical form, solubilization efficiency and percent drug loading as shown in Table 9 [29].

**Table 9:** Stability studies of rosuvastatin-loaded nanogels.

Sr.No	Parameters	0 Month	3^rd^ Month	6^th^ Month
**1**	Physical appearance	Initially light yellow colour	No prominent changes	No prominent changes
**2**	Solubilizing efficiency	Increased upto 26 fold	No significant variations	No significant variations
**3**	Drug loading (%)	87.31±1.17	88.01±1.54	88.69±0.96

## 5. Conclusion

The aim of the current research was to enhancement of rosuvastatin solubility and its dissolution rate as a major concern of sundry pharmaceutical constituents is poor solubility that has a remarkable influence on their bioavailability as well as clinical effects. The objective was accomplished by developing highly amorphous βCD/PVP-co-poly (AMPS) nanogels by free radical polymerization. Nanogels were proficiently loaded by rosuvastatin. Because there was a considerable difference in solubility between the model drug and the loaded nanogels, polymeric nanogels were successful in improving lipophilic drug solubility. The calculated micrometeric properties indicate the fabricated nanogels have good flow properties making them easy to use with adequate stacking of drug. Physicochemical characterization (FTIR, PXRD, DSC, TGA, SEM, sol-gel, and zeta sizer) confirmed the interaction of RST with βCD nanogels. The prepared formulations show enhanced drug loading percentage upto 88%. The drug release pattern reveals the drug is released from all the formulations within 5 to 30 mins. Prepared nanogels have high biocompatibility which is confirmed through haemolytic assay and toxicological studies. It is concluded that the biocompatible nanogels are efficitively fabricated with excellent physiochemical properties, high dissolution and improved solubilization making it potential approach for hydrophobic drug delivery.

## Supporting information

S1 Graphical abstract (JPG)
