# Supplementary figures and images for: Development of β-cyclodextrin/polyvinypyrrolidone-co-poly (2-acrylamide-2-methylpropane sulphonic acid) hybrid nanogels as nano-drug delivery carriers to enhance the solubility of Rosuvastatin: An in vitro and in vivo evaluation
Source: PLoS One. 2022 Jan 21;17(1):e0263026. doi: 10.1371/journal.pone.0263026 (PMC8782392; doi:10.1371/journal.pone.0263026)

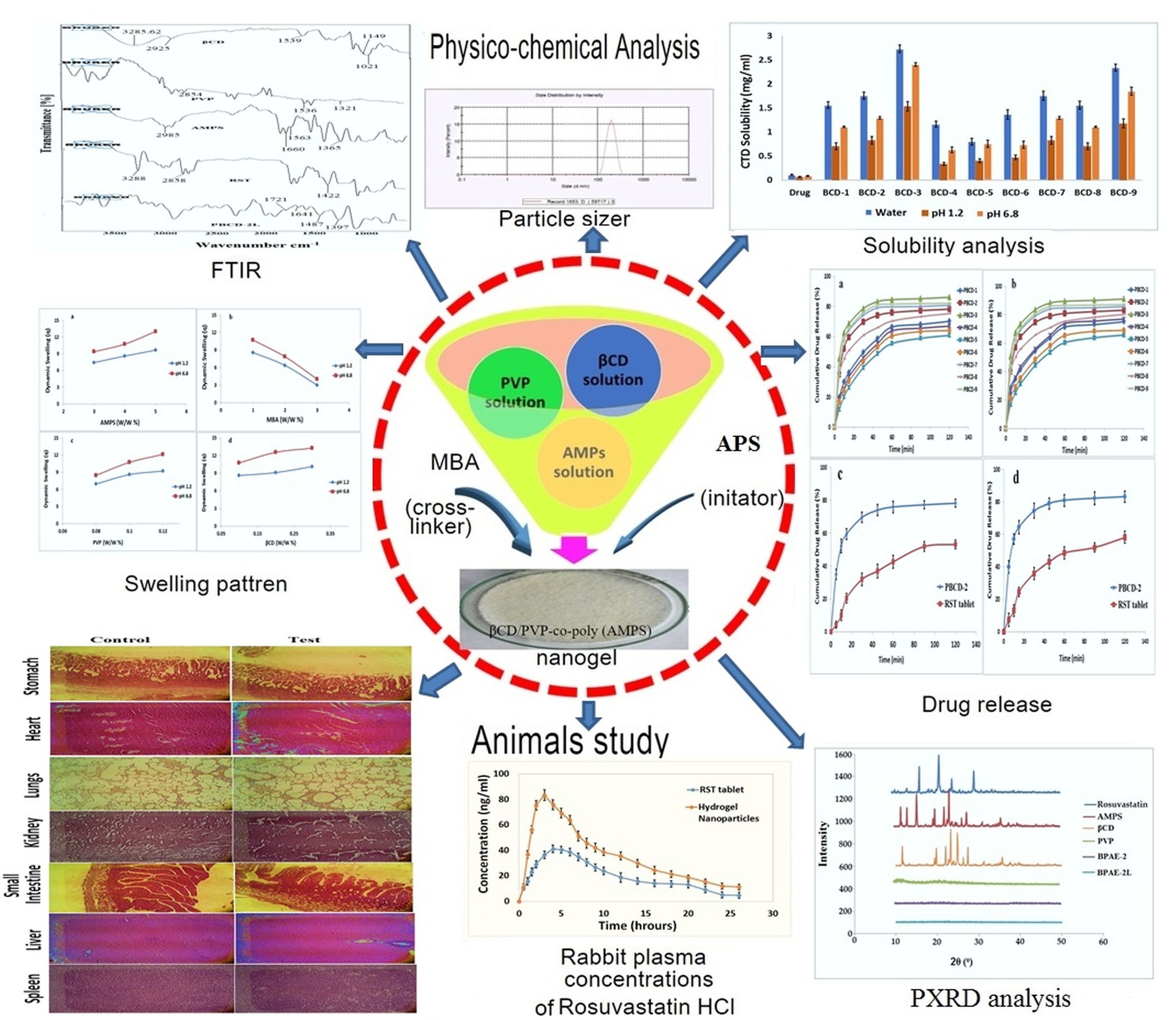

Supplement: S1 Graphical abstract — (JPG) [file pone.0263026.s001.jpg]
